# Neuroprotective Activity of Coptisine from *Coptis chinensis* (Franch)

**DOI:** 10.1155/2015/827308

**Published:** 2015-07-02

**Authors:** Thomas Friedemann, Udo Schumacher, Yi Tao, Alexander Kai-Man Leung, Sven Schröder

**Affiliations:** ^1^HanseMerkur Center for Traditional Chinese Medicine, University Medical Center Hamburg-Eppendorf, Martinistraße 52, 20246 Hamburg, Germany; ^2^Institute of Anatomy and Experimental Morphology, University Medical Center Hamburg-Eppendorf, Martinistraße 52, 20246 Hamburg, Germany; ^3^School of Chinese Medicine, Hong Kong Baptist University, 7 Baptist University Road, Kowloon Tong, Hong Kong

## Abstract

*Coptis chinensis* rhizomes (CR) are one important ingredient of traditional Chinese herbal formulas such as San-Huang-Xie-Xin-Tang which is used for treatment of cardiovascular and neurodegenerative diseases. Recent studies suggest that the extract of CR might be a potential therapeutic agent for amelioration of neurological disorders associated with oxidative stress. In the present study we aimed at revealing the main active compound(s) of the CR extract and at investigating the mechanism of action. Four main alkaloids of the CR extract (berberine, coptisine, jatrorrhizine, and palmatine) were selected for this study. Results showed that out of those alkaloids only pretreatment with coptisine significantly attenuated tert-butylhydroperoxide induced reduction of cell viability, increased rate of apoptosis, and declined mitochondrial membrane potential. Elisa assay and quantitative real-time PCR analyses revealed that thioredoxin-interacting protein (TXNIP) gene expression was downregulated by coptisine, which could explain the neuroprotective effect, hypothetically, by strengthening the thioredoxin defense system against oxidative stress and attenuation of apoptosis signal-regulating kinase (Ask1) mediated apoptotic signaling. A comparison between coptisine and CR extract identified coptisine as the main single component responsible for the neuroprotective effect. Based on the results the CR extract and coptisine are promising candidate agents for prevention or improvement of diabetic neuropathy and neurodegenerative disorders.

## 1. Introduction

*Coptis chinensis* (Franch) rhizomes (CR), commonly known as Coptidis rhizoma or “Huang Lian,” has been used in Traditional Chinese Medicine (TCM) since ancient times and was recommended by famous physicians in TCM history like Li Shizhen, Tang Shenwei, and Tao Hongjing for inflammatory diseases [1].

CR is a main ingredient of multiple historical prescriptions, for example, San-Huang-xie-xin-tang (SHXT), which originated in the Ming dynasty. SHXT is composed of* Coptis chinensis* (Franch) (Coptidis rhizoma),* Rheum officinale* Baill (Rhei rhizoma), and* Scutellaria baicalensis* Georgi (Scutellariae radix) [2]. Recent studies showed that SHXT has neuroprotective properties due to its anti-inflammatory and antioxidative effects [3–5]. It has been shown that one of the main ingredients of SHXT, the dried root CR also known as Chinese goldthread (*huang-lian* in Chinese), is effective for the treatment of neurodegenerative disease associated with oxidative stress [4, 6, 7]. Some of its single compounds showed neuroprotective [8], neuroregenerative [9], anti-apoptotic [10], and antioxidative [6] effects which strongly point out that CR is one main component in decoctions used for the treatment of oxidative stress associated with neurodegenerative disease.

Reactive oxygen species (ROS) have a significant impact on the development of neurodegenerative disease like Alzheimer or Parkinson's disease [11–15]. Cells including neurons are usually well-protected from ROS-induced cytotoxicity by the endogenous antioxidant system. However, if the oxidative stress exceeds the antioxidative capacity of this system it can lead to deoxyribonucleic acid (DNA) demethylation, histone acetylation, oxidative protein and lipid modification, increase of intracellular calcium ions (Ca^2+^), depolarization of the mitochondrial membrane potential (MMP), and release of cytochrome C into the cytosol and, as a consequence, to apoptosis or necrosis [15–19]. For this reason, therapeutic strategies targeting ROS-induced cytotoxicity are needed and could have a major impact on the treatment of neurodegenerative diseases, which are associated with oxidative stress.

We recently showed that 2 h and 24 h pretreatment of SH-SY5Y neuroblastoma cells with the watery extract of CR (CRE) significantly attenuated tert-butylhydroperoxide- (t-BOOH-) induced cytotoxicity [20]. Further statistical analysis revealed that 24 h pretreatment with CRE was more effective than 2 h pretreatment.

The present study aimed at revealing the main active compound of CRE, which is responsible for the cytoprotective effect of the extract, and at comparing the effectiveness of the single compounds with the whole extract.

## 2. Materials and Methods

### 2.1. Drugs and Reagents

tert-Butylhydroperoxide (t-BOOH), thiazolyl blue tetrazolium bromide (MTT), and dimethyl sulfoxide (DMSO) were purchased from Sigma (Taufkirchen, Germany). 2′,7′-Dichlorodihydrofluorescein diacetate (H_2_DCFH-DA), Mitotracker Red CMX Ros, and Hoechst 33342 were obtained from Life Technologies (Darmstadt, Germany). Berberine (Ber), coptisine (Cop), jatrorrhizine (Jat), and palmatine (Pal) were ordered from Cfm Oskar Tropitzsch (Markdredwitz, Germany). All other reagents were purchased from Roth (Karlsruhe, Germany).

### 2.2. Herbal Preparation

*Coptis chinensis *(Franch) was obtained from China Medica (Ch. B. 930034; 83684 Tegernsee, Germany), as dried rhizome. Identity and purity were confirmed according to the Pharmacopoeia of the People's Republic of China [21]. Sebastian Kneipp research laboratory for residue analysis and organic trace analysis (Bad Wörishofen, Germany) certified that heavy metal, pesticide, and microbiological contamination were below the guideline of the Pharmacopoeia Europaea [22] and Regulation (EC) Number 396/2005 of the European Commission.

*Coptis chinensis *extract was prepared as described previously [20]. 10 g of grounded rhizome was boiled in 100 mL distilled deionized water (DDW) for 30 minutes and the extract was centrifuged afterwards. Supernatant was collected and the residue was extracted a second time with 100 mL DDW. Combined supernatants were dried with a rotary-vacuum evaporator (60°C, 200 mbar; Rotavapor-R, Büchi) and a vacuum concentrator (Bachofer). Dried extracts were stored at −20°C until use.

### 2.3. HPLC-Analysis

High-performance liquid chromatography (HPLC) analysis was performed according to the method described previously [20]. Briefly, chromatographic separation was conducted on an Alltima C18 (250 mm × 4.6 mm × 5 *μ*m, S/N: 213100139, temperature: 25°C) column with 0.1% trifluoroacetic acid (A) and acetonitrile (B) as mobile phase and at a flow rate of 1 mL/min. Berberine (Ber), coptisine (Cop), jatrorrhizine (Jat), and palmatine (Pal) were used as reference standard compounds.

### 2.4. Cell Culture

Human neuroblastoma SH-SY5Y cells were cultivated in RPMI 1640 medium containing 10% fetal calf serum, 100 U/mL penicillin, and 100 *μ*g/mL streptomycin. Cells were grown in a humid atmosphere of 5% CO_2_ and 95% air at 37°C. All cell culture reagents were obtained from Sigma (Taufkirchen, Germany).

### 2.5. Cell Viability Measurements

Cell viability was determined by the MTT assay. Ber, Cop, Jat, and Pal stock solutions were diluted in medium to their final concentration and sterilely filtrated, and different concentrations were added at the start of the incubation time for 24 h. Afterwards, cells were incubated for 2 h with 100 *μ*M t-BOOH. Medium containing t-BOOH was removed, cells were washed with Dulbecco's phosphate-buffered saline (DPBS), and 1 mM MTT solution was added for 2 h. Subsequently, 100 *μ*L 2-Propanol was added and the plate was agitated for 1 h at 450 revolutions per minute (rpm) at room temperature (RT). Absorption was measured 3 times at 570 nm.

### 2.6. Quantification of ROS

Reactive oxygen species in the cells were measured with the 2′,7′-dichlorodihydrofluorescein diacetate probe (H_2_DCFH-DA). SH-SY5Y cells were seeded into a 96-well microplate (4*∗*10^4^ cells/well) and incubated with 100 *μ*g/mL CRE or 20 *μ*M Cop for 24 hours. Afterwards, fresh medium containing 20 *μ*M H_2_DCFH-DA was added for 30 min at 37°C in the dark. Subsequently, cells were washed with DPBS and medium containing 100 *μ*M t-BOOH was added. Fluorescence was measured every 10 minutes for 120 minutes (excitation: 485 nm; emission: 528 nm).

### 2.7. Quantification of Apoptotic Nuclei and Mitochondrial Membrane Potential

Apoptotic nuclei and mitochondrial membrane potential were measured using Mitotracker Red CMX Ros and Hoechst 33342. Cells were stained first with 25 nM Mitotracker Red CMX Ros for 1 hour, followed by 20 minutes fixation with 4% paraformaldehyde (PFA) and 10 minutes Hoechst 33342 (4 *μ*M) staining. Images were captured with a Leica microscope and an Axiovision camera. To determine MMP, the intensity sum of the Mitotracker Red CMX Ros fluorescence was measured for each cell. Apoptosis was detected by analyzing the morphology of the Hoechst 33342 stained nuclei. Experiments were repeated 3 times and at least 1600 cells were analyzed for each group.

### 2.8. RT-PCR

Total RNA was isolated with the RNeasy MINI Kit (Quiagen; Hilden; Germany) according to the manufacturer's instructions. Reverse transcription was carried out with the high capacity RNA-to-cDNA Kit (Applied Biosystems). For semiquantitative analysis, LightCycler 480 SYBR Green 1 Master (Roche) and the following human specific primers were used: TXNIP 5′-GATCACCGATTGGAGAGCCC-3′ and 5′-TGCAGGGATCCACCTCAGTA-3′; GAPDH 5′-GCATCTTCTTTTGCGTCGCC-3′ and 5′-CCCAATACGACCAAATCCGTTG-3′. Obtained data were analyzed as previously described [23].

### 2.9. Human TXNIP Elisa Assay

Cells were seeded in T-25 flasks (2.5 × 10^6^ cells/flask) and treated for 24 hours with CRE or Cop. Total proteins were isolated with mammalian cell lysis reagent (CellLytic M) according to the manufacturer's instructions and stored at −80°C until use.

Protein concentration was determined with Roti-Quant following the protocol provided by the manufacturer. Human TXNIP protein concentration was measured with the CircuLex Human TXNIP ELISA Kit (MBL international cooperation, Biozol, Germany) following the instructions of the provided protocol. Experiments were repeated three times and each sample was measured in duplicates.

### 2.10. Statistics

Data are presented as means ± standard error of the mean (SEM) of *n* experiments. Statistical significance between groups was determined with OriginLab pro 8.5 by ANOVA, followed by the Bonferroni Post Hoc Test. *P* < 0.05 was considered as statistically significant.

## 3. Results

### 3.1. HPLC Analysis

**Figure 1:**
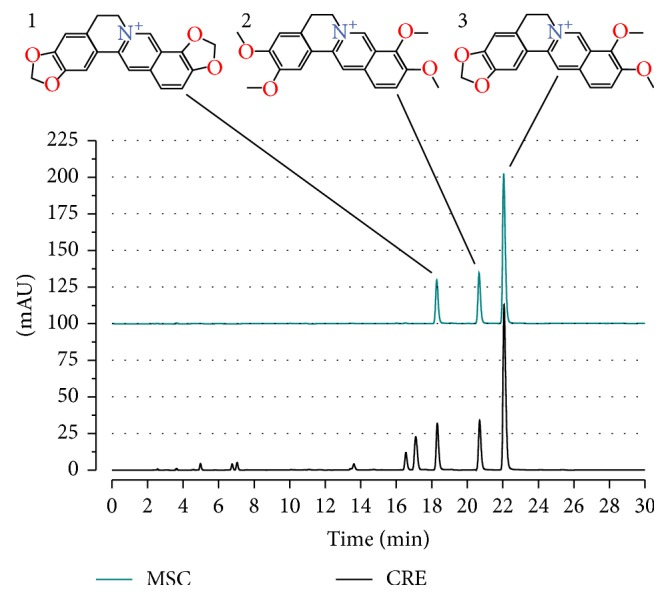
High-performance liquid chromatogram of the watery extract from* Coptis chinensis* (Franch). Blue line represents the HPLC chromatogram of mixed standard compounds (MSC); 1: coptisine, 2: palmatine, 3: berberine. Note that coptisine, berberine, and palmatine are the main components of the CRE. ACD/ChemSketch (http://www.acdlabs.com/resources/freeware/chemsketch/) was used to design chemical structures.

Watery extraction of CR yields 21.5% by weight of the dried herb. The CRE was composed of 554.9 ± 14.6 mg/g Ber, 60.6 ± 0.5 mg/g Cop, and 51.9 ± 0.2 mg/g Pal. Representative high-performance liquid chromatogram of the extract and of the mixed standard compounds is shown in Figure 1.

### 3.2. Effect of CRE and CR Main Alkaloids on Cell Viability

**Figure 2:**
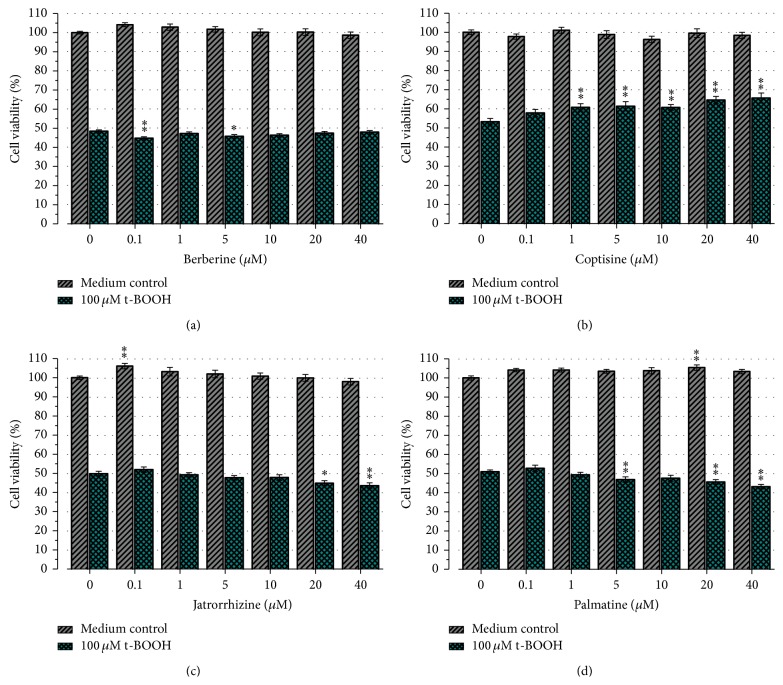
Effect of CR main alkaloids on cell viability in SH-SY5Y cells. Cells were pretreated for 24 h with (0–40 *μ*M) of Ber (a), Cop (b), Jat (c), and Pal (d) before the cells were exposed for 2 h to either medium (medium control) or 100 *μ*M t-BOOH (t-BOOH control). Results represent mean cell viability ± SEM of four independent experiments conducted in triplicates. Only treatment with Cop resulted in a significant increase of cell viability. ^*∗*^ *P* < 0.05, ^*∗∗*^ *P* < 0.01 versus medium or t-BOOH control.

To investigate the neuroprotective effect of CR main alkaloids against t-BOOH-induced toxicity, we first examined if those single components exhibited any cytotoxic effect in the concentration range between 0.1 and 40 *μ*M. Results showed no significant cytotoxic effect of Ber, Cop, Jat, and Pal in comparison to the medium control (*P* < 0.01, Figure 2). Treatment of cells with 100 *μ*M t-BOOH resulted in significant decrease of cell viability to 53.2 ± 1.7% (*P* < 0.01; versus medium control). Pretreatment of the cells with Ber, Jat, and Pal (0.1–40 *μ*M) before t-BOOH-induced oxidative stress showed no significant protective effect compared to the t-BOOH control (Figures 2(a), 2(c), and 2(d)). However, pretreatment with 1–40 *μ*M Cop for 24 hours resulted in a significant increase of cell viability from 53.2 ± 1.7% (t-BOOH control) up to 65.6 ± 2.6% (40 *μ*M, Figure 2(b)).

**Figure 3:**
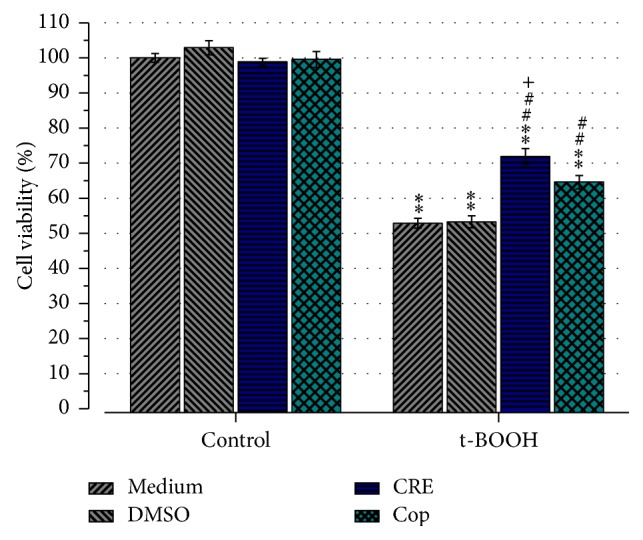
Effect of CRE and Cop on cell viability in SH-SY5Y cells. Cells were pretreated for 24 h with CRE (100 *μ*g/mL) or Cop (20 *μ*M). Note that treatment with Cop achieves 80% of the effect of CRE. ^*∗∗*^ *P* < 0.01 versus medium or DMSO control. ^##^ *P* < 0.01 versus t-BOOH-medium or t-BOOH-DMSO. ^+^ *P* < 0.05 versus t-BOOH-Cop.

Previously, we showed that 24-hour pretreatment with the watery extract of CR had a significant protective effect against t-BOOH-induced oxidative stress, whereas 100 *μ*g/mL was most effective [20]. To investigate if Cop is responsible for the previously reported protective effect of CRE, we compared 24-hour pretreatment of CRE (100 *μ*g/mL) with Cop (20 *μ*M). Results revealed that CRE increased cell viability to 71.9 ± 2.3% and Cop to 64.6 ± 1.9% (Figure 3). Statistical analyses showed that CRE is significantly more effective than Cop (*P* < 0.05). However, 80% of the cell viability increase of CRE could be achieved by Cop (Figure 3).

### 3.3. Effect of CRE and Cop on Intracellular Reactive Oxygen Production

**Figure 4:**
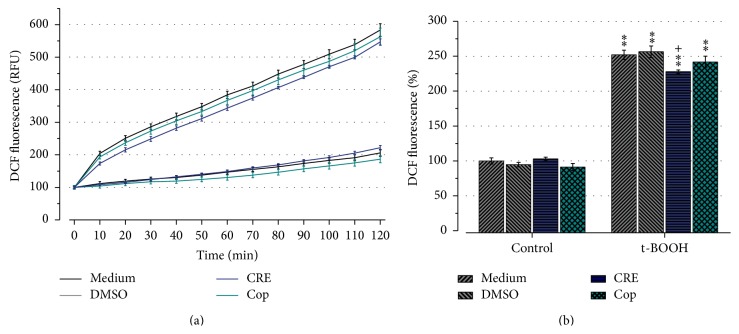
Effect of CRE (100 *μ*g/mL) and Cop (20 *μ*M) on DCF fluorescence was investigated in SH-SY5Y cells. Increase in DCF fluorescence indicates increased intracellular ROS. (a) shows the evolution of ROS generation; t-BOOH (upper 4 lines) or PBS (lower 4 lines) was added at the start of the measurements. Results are expressed as mean DCF fluorescence ± SEM normalized to the medium control of three independent experiments which were conducted in triplicate. (b) represents the mean sum of the DCF fluorescence signal normalized to the medium control, which was set to 100%. Note that only pretreatment with CRE significantly reduces mean DCF fluorescence. ^*∗∗*^ *P* < 0.01 versus control. ^+^ *P* < 0.05 versus Cop.

To investigate the antioxidative effect of CRE and Cop, we analyzed the production of intracellular reactive oxygen species induced by t-BOOH treatment. The mean dichlorofluorescein (DCF) fluorescence of medium control cells was set to 100%. Results showed that both CRE and Cop had no effect on the baseline DCF fluorescence. Two-hour exposure to 100 *μ*M t-BOOH produced a significant increase in DCF fluorescence to 252.1 ± 6.7% (*P* < 0.01; t-BOOH control). Furthermore, results revealed that only CRE significantly reduced mean DCF fluorescence to 227.7 ± 2.5% (*P* < 0.5 versus t-BOOH control, Figure 4).

### 3.4. Effect of CRE and Cop on Apoptotic Rate

**Figure 5:**
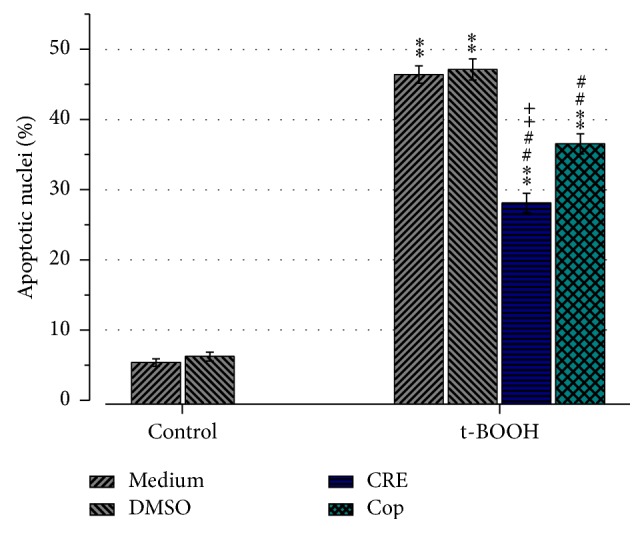
Effect of CRE (100 *μ*g/mL) or Cop (20 *μ*M) on t-BOOH-induced apoptosis. Results represent the percentage of apoptotic nuclei ± SEM from the total number of counted nuclei. 3 independent experiments were conducted and 1600 cells were analyzed at least in each group. Results showed that both COP and CRE significantly attenuate t-BOOH-induced apoptosis. ^*∗∗*^ *P* < 0.01 in comparison to the control (no treatment); ^##^ *P* < 0.01 versus t-BOOH-medium or t-BOOH-DMSO and ^++^ *P* < 0.01 versus t-BOOH-Cop.

In order to investigate the effect of CRE and Cop on t-BOOH-induced apoptosis we analyzed the morphology of the nucleus. Results showed that the apoptotic rate in both medium and DMSO control groups was 5.3 ± 0.5% and 6.2 ± 0.6%, respectively. Two hours of t-BOOH treatment increased apoptotic rate in the medium and DMSO control group significantly to 46.3 ± 1.2% and 47.1 ± 1.5% (*P* < 0.01, Figure 5). Twenty-four hour pretreatment with CRE (100 *μ*g/mL) or Cop (20 *μ*M) reduced apoptosis significantly to 28.0 ± 1.3% and 36.5 ± 1.4%, respectively (*P* < 0.01). Statistical analysis revealed that CRE attenuates t-BOOH-induced apoptosis significantly more than Cop (*P* < 0.05).

### 3.5. Effect of CRE and Cop on Mitochondrial Membrane Potential

**Figure 6:**
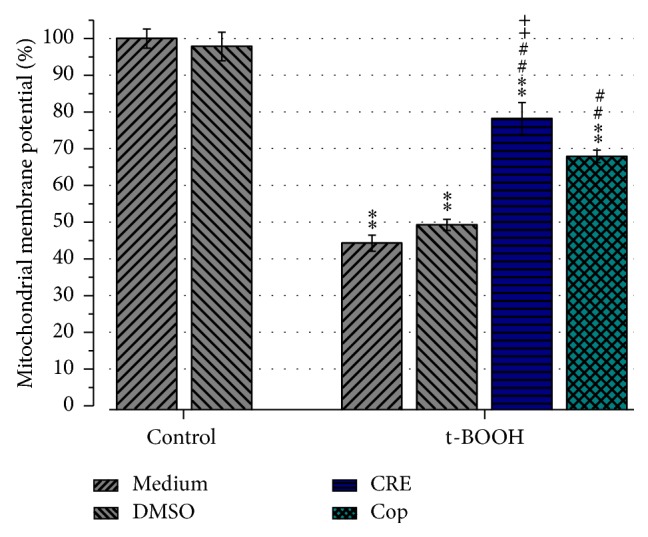
Effect of 24 h pretreatment with CRE (100 *μ*g/mL) and Cop (20 *μ*M) on mitochondrial membrane potential. Pretreated cells were challenged with oxidative stress induced by 2 h t-BOOH treatment. The mean MMP ± SEM was normalized to the medium control which was set to 100%. Experiments were repeated twice and for each group the MMP of 1600 cells were measured at least. Pre-treatment of the cells with CRE and Cop significantly increased MMP compared to the t-BOOH-medium control. ^*∗∗*^ *P* < 0.01 versus control; ^##^ *P* < 0.01 versus t-BOOH-medium or t-BOOH-DMSO and ^++^ *P* < 0.01 versus t-BOOH-Cop.

Since it was previously reported that oxidative stress induced by t-BOOH leads to opening of the mitochondrial permeability transition (MPT) pore and loss of mitochondrial membrane potential [18, 19, 24], we investigated if CRE or Cop could attenuate the loss of mitochondrial membrane potential (MMP) in SH-SY5Y cells. Results showed that 2-hour exposure of SH-SY5Y cells with 100 *μ*M t-BOOH significantly reduced the fluorescence signal to 44.2 ± 2.1% (t-BOOH-medium control) and 49.2 ± 1.5% (t-BOOH-DMSO control) in comparison to the medium control (*P* > 0.01), which was set to 100% (Figure 6). Pretreatment of the cells for 24 hours with 100 *μ*g/mL CRE or 20 *μ*M Cop significantly increased the MMP compared to the t-BOOH medium control to 78.2 ± 4.3% and 67.9 ± 1.7%, respectively (*P* < 0.01). Comparison between the CRE and Cop group showed that CRE was significantly more effective than Cop (*P* > 0.05).

### 3.6. Effect of CRE and Cop on TXNIP Expression

**Figure 7:**
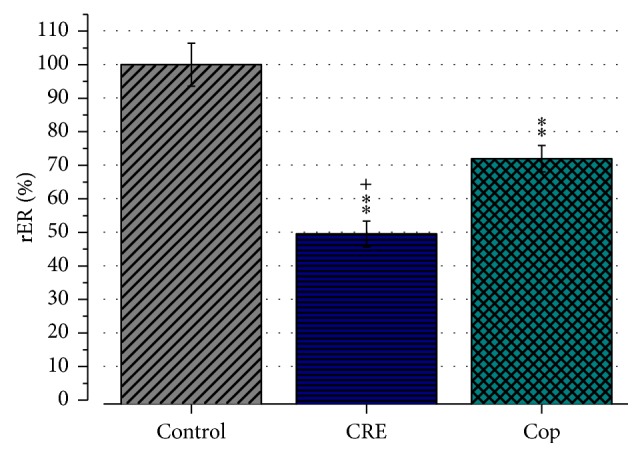
Effect of CRE (100 *μ*g/mL) and Cop (20 *μ*M) on TXNIP gene expression. Results represent mean relative gene expression ratio (rER) ± SEM. Experiments were performed in triplicates and repeated twice. Data were normalized to the nontreatment control which was set to 100%. Note that treatment of the cells with CRE or Cop leads to a significant downregulation of TXNIP. *∗∗* indicates *P* < 0.01 versus control. ^+^ *P* < 0.05 versus Cop.

QRT-PCR results confirmed that treatment of the cells with CRE or Cop leads to a significant downregulation of TXNIP compared with the control group to 49.5 ± 3.8% and 71.9 ± 3.9%, respectively (*P* < 0.01; Figure 7). A comparison between CRE and Cop groups revealed that the downregulation of TXNIP was significantly pronounced in the CRE group (*P* < 0.05).

**Figure 8:**
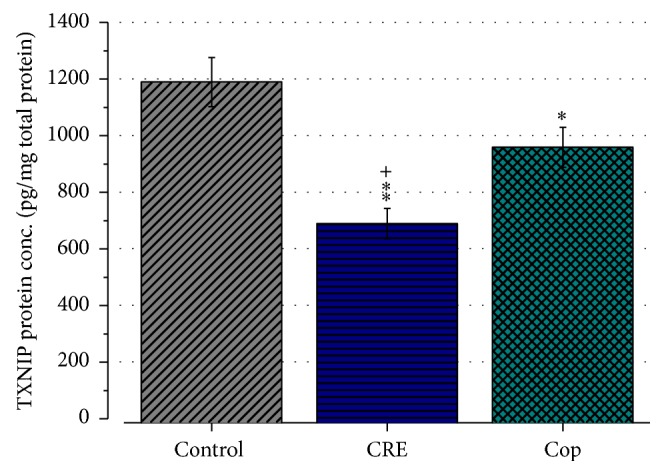
TXNIP protein concentration of 1 mg extracted total protein. Results represent mean TXNIP concentration ± SEM of three independent experiments preformed in duplicates. TXNIP protein concentration was significantly reduced by 24 h CRE (100 *μ*g/mL) and Cop (20 *μ*M) treatment. ^*∗*^ *P* < 0.05, ^*∗∗*^ *P* < 0.01 in comparison to the control; and ^+^ *P* < 0.05 versus Cop.

Furthermore, results of the TXNIP protein expression revealed that the TXNIP concentration in the control, CRE, and Cop group was 1189.2 ± 86.6 pg/mg total protein, 689.4 ± 53.2 pg/mg total protein, and 958.7 ± 70.4 pg/mg total protein, respectively (Figure 8). Statistical analysis showed that both CRE and Cop reduced the TXNIP protein concentration significantly when compared to the control (*P* < 0.01 and *P* < 0.05) and that CRE was significantly more effective than Cop (*P* < 0.01).

## 4. Discussion

In recent years many studies focused on cellular injuries related to oxidative stress and natural antioxidants with neuroprotective potential. A plurality of the investigated natural herbal extracts or single compounds exert their protective effect via removal of surplus ROS or by the prevention of ROS generation. However, it turned out to be that many natural antioxidants, even if they show some beneficial effect in cell culture experiments, have no or just limited effect in humans [25]. Therefore, other natural pharmaceuticals which act via a different mechanism to protect the cells from oxidative damage are needed.

In this study we investigated the neuroprotective effect against t-BOOH-induced oxidative damage of the main compounds of CR, a very important herb in Chinese medicine, which is used in many different herbal prescriptions.

Our results confirmed that 2-hour treatment of SH-SY5Y cells with t-BOOH resulted in a significant cell viability reduction with a strong dose-dependency. There was no attenuation of the cell viability loss detectable by pretreatment with different concentrations of Ber, Jat, and Pal. However, pretreatment with Cop resulted in a dose dependent reduction of t-BOOH-induced cytotoxicity by improvement of the mitochondrial membrane potential and attenuation of apoptosis. A comparison with the cytoprotective effect of CRE revealed that pretreatment with Cop in a comparable concentration, as in the CRE, achieved approximately 80% of the effect of the CR watery extracts. This suggests that Cop alone is not the only compound responsible for the neuroprotective effect; however, it is the main active compound. It has been shown previously that the extract of Coptis chinensis includes the following compounds: magnoflorine, groenlandicine, berberastine, demethyleneberberine, lycoranine B, jatrorrhizine/columbamine, epiberberine, coptisine, thalifendine/berberrubine, palmatine, berberine and dihydrochelerythrine [26–29]. In this study, we used only 4 alkaloids of the extract, namely, berberine, coptisine, jatrorrhizine, and palmatine. Therefore, we hypothesize that the cytoprotective effect of CR against t-BOOH-induced oxidative stress could be imitated by the combination of two or more single compounds.

It was previously reported by Drahota et al. that treatment with t-BOOH leads to a reduction of the MMP and increases apoptosis in vitro [30]. Therefore, we tested if Cop pretreatment attenuates the reduction of the MMP as well as the increased apoptotic rate and compared the results with the effect of the CRE. Our results revealed that pretreatment of SH-SY5Y cells with Cop or CRE improved the MMP compared to the t-BOOH control and that the CRE was significantly more effective than Cop. The same finding also applies to the reduction of t-BOOH-induced apoptosis by Cop and CRE. Furthermore, these findings strengthen the hypothesis that the cytoprotective effect of the CRE could only be explained by a combination of different individual compounds. Nonetheless, Cop seems to be the main active component which protects the cells against t-BOOH-induced oxidative stress.

Based upon these results, it is suggested that the cytoprotective effect is at least partly achieved by improved mitochondrial function and reduction of apoptosis.

To verify if the neuroprotective effect of CRE and COP against t-BOOH-induced oxidative stress could be explained furthermore by the antioxidative properties of the extract we investigated the antioxidative effect of CRE and Cop with the cell-based H_2_DCFH-DA assays. The results showed that only the CRE showed a slight antioxidative effect. However, this effect was too small to explain the present neuroprotective effect of the CRE. The results suggest that the amount of antioxidative compounds in the extract or the concentration of Cop was too low to have a significant influence on the ROS generation induced by the rather high dose of t-BOOH.

We previously reported that genome-wide transcriptome analysis on Human Genome U219 microarrays (Affymetrix, SantaClara, USA) of SH-SY5Y cells revealed that 24 h treatment with CRE significantly regulated only the expression of two genes: mitochondrially encoded NADH dehydrogenase 1 (MTND1) and TXNIP [20]. In this work we hypothesized that downregulation of TXNIP, a 50-kDa protein which belongs to the *α*-arrestin protein family, could be at least partly responsible for the neuroprotective effect of CRE on t-BOOH-induced oxidative damage. It is well-known that TXNIP is an inhibitor of thioredoxin (Trx), which is essential for intracellular responses to ROS [31–33]. In case of oxidative stress TXNIP binds to Trx, which prevents Trx in playing its major role as antioxidant, in DNA synthesis and repair, in cellular signaling and transcription control, and in inhibition of apoptotic pathways [31–36].

Therefore, downregulation of TXNIP by CRE or Cop could reduce the amount of inactivated Trx, resulting in an attenuation of apoptotic signaling in the cell [37, 38]. This suggests that downregulation of TXNIP could protect SH-SY5Y cells from apoptosis, improve DNA repair as well as cellular signaling, and strengthen oxidative defense system during high oxidative stress. Our results revealed that both CRE and Cop decreased TXNIP, mRNA, and protein concentration. The reduction of TXNIP, at least for Cop, seems to be too low to explain the present neuroprotective effect of Cop on t-BOOH-induced oxidative stress. However, the reduction of TXNIP expression should have an impact on cellular ROS level. Yoshihara et al. reported that downregulation of TXNIP attenuates the inhibition of the Trx redox cycle by TXNIP and this results in a reduction of cellular oxidative stress [39]. Since our results showed only a small reduction of ROS by CRE and no effect of Cop, one might speculate that the present downregulation of TXNIP was not sufficient enough to have a significant influence on the ROS level generated by the relatively high t-BOOH concentration used in this study. Gao et al. showed that inhibition of TXNIP expression by activation of AMP-activated protein kinase protects podocytes from oxidative stress induced injury, without any influence of the cellular ROS level [38]. They hypothesized that a reduction of TXNIP expression leads to an attenuation of the ASK1-P58 signaling pathway which protects the cells from oxidative stress induced injuries. Nevertheless, further studies should clarify if downregulation of TXNIP by CRE and Cop also leads to a attenuation of the ASK1-P58 signaling pathway in SH-SY5Y cells and whether this mechanism is involved in the neuroprotective effect of CRE and Cop against oxidative stress induced cytotoxicity.

## 5. Conclusion 

In conclusion, we showed that pretreatment of SH-SY5Y neuroblastoma cells with Cop attenuated t-BOOH-induced decrease in cell viability, reduction of the MMP, and increase of apoptosis. Further analysis showed that the protective effect of CRE against t-BOOH-induced cytotoxicity was more pronounced than the effect of Cop. Nonetheless, we could achieve approximately 80% of the effectiveness of CRE by treatment with Cop. This leads us to the conclusion that Cop is the main active single component of CRE for the prevention of t-BOOH-induced oxidative stress cell injury and that the protective effect might be based on an improvement of the mitochondrial function as well as on the attenuation of proapoptotic pathways, hypothetically, the ASK1-P58 signaling pathway.

This research work provided evidence that CRE and Cop might have potential therapeutic value for the treatment of neurodegenerative diseases associated with oxidative stress, like Alzheimer's disease or Parkinson's disease.

## Abbreviations

Ask1: Apoptosis signal-regulating kinase
Ber: Berberine
Ca^2+^: Calcium ion
Cop: Coptisine
CR: * Coptis chinensis* rhizomes
CRE: * Coptis chinensis* rhizomes watery extract
DCF: Dichlorofluorescein
DDW: Distilled deionized water
DMSO: Dimethyl sulfoxide
DNA: Deoxyribonucleic acid
DPBS: Dulbecco's phosphate-buffered saline
H_2_DCFH-DA: 2′,7′-Dichlorodihydrofluorescein diacetate
HPLC: High-performance liquid chromatography
Jat: Jatrorrhizine
MMP: Mitochondrial membrane potential
MSC: Mixed standard compounds
MTT: Thiazolyl blue tetrazolium bromide
Pal: Palmatine
PFA: Paraformaldehyde
qRT-PCR: Quantitative real-time-PCR
ROS: Reactive oxygen species
rpm: Revolutions per minute
RT: Room temperature
SEM: Standard Error of the Mean
SHXT: San-Huang-xie-xin-tang
t-BOOH: tert-Butylhydroperoxide
TCM: Traditional Chinese Medicine
Trx: Thioredoxin.
